# Recovery Trends of Commercial Fish: The Case of an Underperforming Mediterranean Marine Protected Area

**DOI:** 10.1371/journal.pone.0146391

**Published:** 2016-01-07

**Authors:** Stefano Marra, Stefania Coppa, Andrea Camedda, Carlotta Mazzoldi, Francesco Wrachien, Giorgio Massaro, G. Andrea de Lucia

**Affiliations:** 1 Institute for Coastal Marine Environment-National Research Council (IAMC-CNR), Oristano, Italy; 2 Department of Ecology and Biology, University of Tuscia, Viterbo, Italy; 3 Department of Biology, University of Padua, Padua, Italy; 4 Marine Protected Area “Penisola del Sinis-Isola di Mal di Ventre”, Cabras, Italy; Hellenic Centre for Marine Research, GREECE

## Abstract

Temporal trends in the recovery of exploited species in marine protected areas (MPAs) are useful for a proper assessment of the efficacy of protection measures. The effects of protection on the fish assemblages of the sublittoral rocky reefs in the “Penisola del Sinis-Isola di Mal di Ventre” MPA (W. Sardinia, Italy) were evaluated using a multi-year series of data. Four surveys, conducted 7, 10, 13 and 15 years after the area was designated as an MPA and carried out in the period spanning June and July, were used to estimate the abundance and biomass of commercial species. The surveys were carried out in zones with decreasing levels of fishing restrictions within the MPA (zones A, B, C) and in unprotected zones (OUT1 and OUT2), and underwater video visual census techniques were used. Protected zones only occasionally showed higher levels of abundance or biomass, and the trajectories of those metrics were not consistent across the years. In addition, the zone with the highest level of protection (zone A) never presented levels of abundance and biomass higher than those in zones B and C. This study shows that even 15 years after designation, protection has had no appreciable effect in the MPA studied. It is argued that this is emblematic of several shortcomings in the planning, regulation and enforcement frameworks of the MPA.

## Introduction

Fishing pressure is considered one of the main sources of impact on marine ecosystems, causing the fragmentation of habitats, alteration of marine communities and a loss of biodiversity and ecosystem functions [1–4]. Recent literature contains many case studies of over-exploitation of marine species and decline of commercial stocks [5], and there is growing evidence that marine communities may be seriously impacted not only by commercial fishing, but also by recreational fishing [6–8]. Many marine protected areas (MPAs) have been established in recent years: the principal reason is that they were believed to be effective tools for contrasting the effects of overfishing [9], although they are more than mere fishing management tools [10]. There is an abundance of literature on the effectiveness of MPAs as conservation tools for marine biological resources [11–14], and an increase in the species richness of marine communities, the greater abundance and mean size of target species in protected sites than in unprotected areas are usually considered indicative of the so-called reserve effect [15–17]. The various types of MPAs include no-take reserves—where fishing is strictly banned throughout—and partially protected areas (PPAs), where some fishing activities are allowed. Mediterranean MPAs are usually established as “multiple-use marine reserves”, i.e. comprising both no-take/no-entry zones and PPAs, which are defined as “buffer zones” [18]. The advantage of these buffer zones is that they are able to reconcile habitat conservation objectives with social and economic demands [19], thus reducing conflict between the stakeholders involved. Most of the Mediterranean MPAs are in coastal areas, where human pressure on marine habitats is higher and where different types of fisheries exist [20, 21]. Therefore, there is a trade-off between the designation of marine reserve to protect marine biological resources and the will to preserve the local fishing economy and those social activities that require access to marine ecosystems [22]. Although PPAs may increase local compliance with MPA management rules, there is considerable debate over the efficiency of protection measures of this kind for recovering exploited stocks [23–25]: in buffer zones, the response to protection measures is less intense than in no-take zones [25], and partially protected assemblages may not differ significantly from unprotected assemblages [26–29]. Therefore, a proper assessment of the effectiveness of a multiple-use MPA as a conservation tool from the fisheries perspective must take account not only of how the characteristics of the marine community in the MPA differ from the characteristics of the unprotected sites (reserve effect), but also of the differences in the response to protection between one protection zone and another (zonation effect).

Most studies have considered the effects of protection on fish, and have focused specifically on shallow assemblages [11]. Shallow fish assemblages (up to 10 metres in depth) are more exposed to human pressure, and thus show stronger responses to protection than assemblages from deeper habitats [30]. Many studies on MPAs report results from single monitoring surveys [12], but there is on-going evidence that only multi-year series of data allow for a proper assessment of the effectiveness of protection measures [31]. Long-term studies on the effect of protection on both fish and marine invertebrates indicate that the response to protection may vary in magnitude, direction and consistency over time [15, 16, 32]. The time required to respond to a reduction in fishing pressure depends on the life history, trophic group, mobility and level of exploitation of the various species [33], and in some cases, this response may become evident only after lengthy periods of protection [34–37]. However, in most fish and invertebrate target species, the direct effects of protection become evident quickly, usually occurring within five years after the creation of a MPA [32, 38]. With regard to fish, the dusky grouper (*Epinephelus marginatus*), for instance, is a long-life fish species with a slow growth rate, and therefore requires about fifteen years of protection (e.g. [39, 40]) or more [41] before a significant increase in abundance can be observed subsequent to the implementation of fishing restrictions. On the other hand, in sea breams (*Diplodus* spp.), which have faster growth rates, a response to protection measures may be evident after just 3 years [42] or 5 years [43].

In practice, however, MPAs do not always prove effective for the conservation of biological resources, and some are merely “paper parks”, i.e. conservation areas that exist solely on paper. In Italy, for instance, there are many MPAs where the effects of protection are not evident [44]. There may be several reasons for low performance: MPAs may fail to meet their management objectives because of inappropriate planning, ineffective enforcement and/or poor acceptance by local communities [45–47]. For example, if the local community does not comply with the regulations of the MPA, illegal fishing is more likely to occur, thus diminishing the MPA’s ability to protect biological resources. If protection of resources is perceived as inefficient, (e.g. there is no improvement in the fauna within the protected area), negative perceptions of the MPAs are likely to increase locally, thus producing a negative feedback loop. Effective monitoring of the effectiveness of MPAs is therefore extremely important, since ineffective protection may render the MPAs less useful and thus negatively impact acceptance on the part of local communities. For a clearer understanding of whether protection measures work in practice, the temporal trends in the recovery of exploited species should be evaluated, but this is not always possible, because of the lack of data from long-term monitoring surveys. In Sardinia (Italy, W. Mediterranean Sea), several MPAs were established by the Italian Ministry of Environment at the end of the 1990s, under National Law no. 394 of 6/12/1991. The “Penisola del Sinis-Isola di Mal di Ventre” Marine Protected Area (hereafter Sinis MPA) is a publicly owned MPA, designated in 1997. No quantitative data regarding the status of local marine resources before the establishment of the MPA were available, and the first survey to monitor the fish fauna was conducted seven years after the establishment of the MPA.

The aim of this study is to address the efficacy of the different degrees of protection and the temporal trends in the recovery of fish assemblages in the Sinis MPA after fifteen years of protection, using a multi-year series of data from four monitoring surveys conducted between 2004 and 2012. Specifically, in accordance with the findings from long-term studies on the effectiveness of MPAs (e.g. [11], and literature cited therein), the following hypotheses were tested: 1) the abundance and biomass of commercial species are higher in zones with the strictest fishing restrictions, with maximum levels present in zone A, intermediate levels in buffer zones, and minimum levels in unprotected zones; 2) the difference in the abundance and biomass of commercial species among different protection zones and unprotected localities tends to increase over the years.

## Materials and Methods

### Ethics statement

No endangered species were involved in this study, and no specimens or any other kind of samples were collected, handled or injured. The data reported in this paper were obtained exclusively from video records taken in the field, using a non-destructive technique (see “Data Collection” section for details). The results reported in this study come from Institute for Coastal Marine Environment surveys commissioned by the “Penisola del Sinis-Isola di Mal di Ventre” MPA and funded by the Italian Ministry of the Environment (Grant Agreements No: DPN/2006,DPN/2009/0016436) and the Regional Environmental Action Plan (P.O.R. FESR 2007–2013, Axis IV; Grant Agreements No. 170/AM).

### Study area

**Fig 1:**
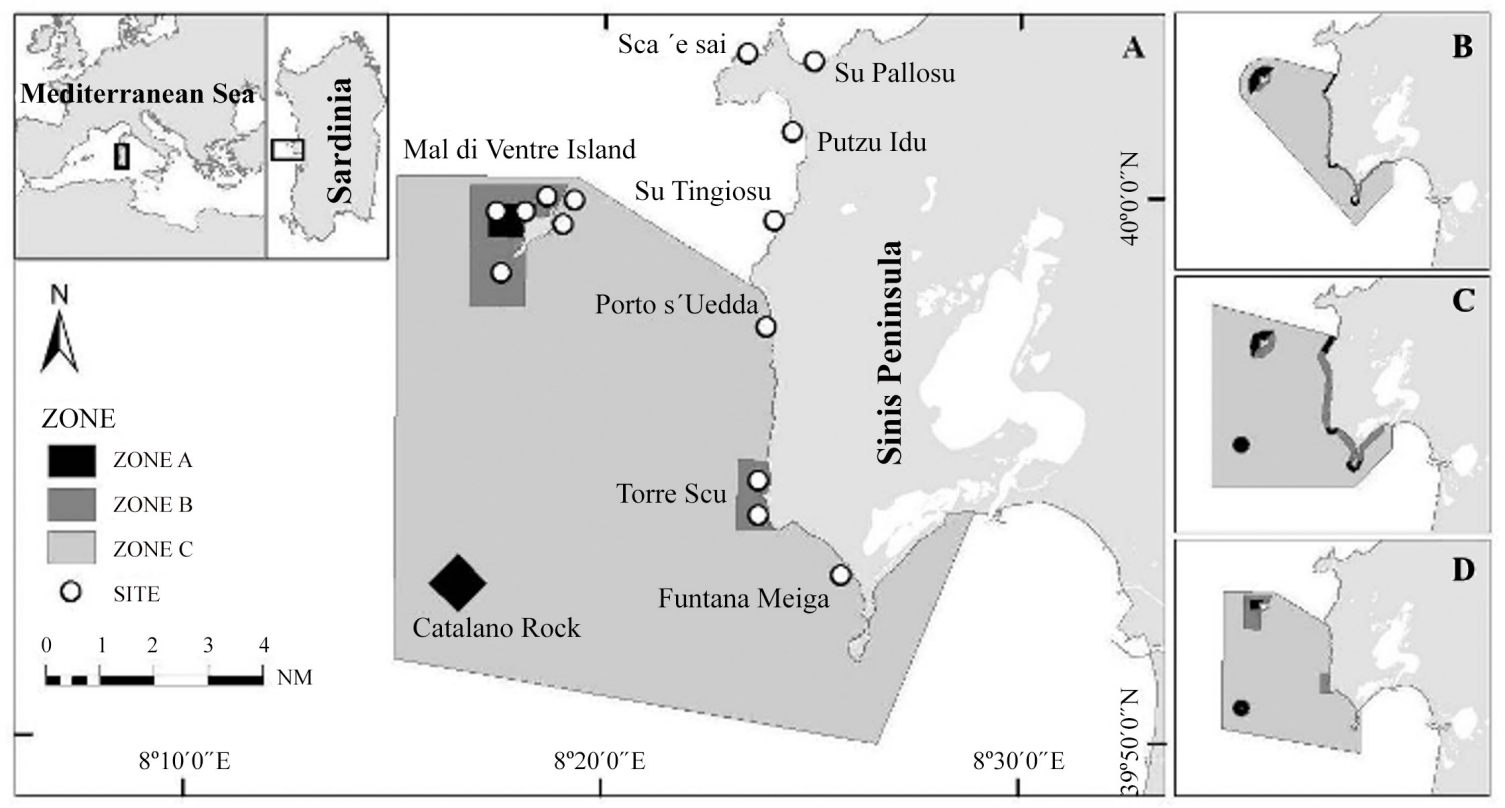
Location and zonation of the “Penisola del Sinis-Isola di Mal di Ventre” Marine Protected Area with the sampling sites considered. Zone A: no-take/no-entry area; zone B: general protection area; zone C: partial protection area. The boxes show the changes in zonation in the Sinis MPA since designation: (A) Current zonation, in force since 2011. (B) Zonation proposed before the MPA was established. (C) Zonation adopted when the MPA was established in 1997. (D) Zonation adopted in 2003.

The Sinis MPA (Fig 1) is on the Central West Coast of Sardinia and is the second-largest marine protected area in Italy (~25000 ha). It is subdivided into three zones, with different degrees of restrictions on human activities: in the no-take/no entry zone (zone A, according to Italian legislation), only surveillance and scientific research are permitted, in the "general protection zone" (zone B) and in the "partial protection zone" (zone C), commercial and recreational fishing are permitted, although the number of authorised fishermen is restricted. The difference between zones B and C is minor, and only regards the number of anglers authorised to fish in the two zones. While in zone B only local fishermen are allowed, in zone C non-residents can also practise recreational angling (Table 1). However, no data were available in the timeframe of this study to quantify this difference. The zonation of the MPA has changed several times over the years, due to mediation designed to reconcile the conflicting interests of stakeholders. Specifically, in 2003, zone B, which once included most of the coastline, was significantly reduced, and three no-take/no-entry areas were removed; one of them (Su Tingiosu) was entirely excluded from the MPA, and is one of the control locations used in this study (Fig 1).

**Table 1:** List of the activities permitted in the different protection zones of the Sinis MPA.

	A	B	C	OUT
**Size (ha)**	358	975	22917	-
**Activities**				
Surveillance; scientific research	+	+	+	+
Bathing	-	+	+	+
Boating; mooring	-	+	+	+
Diving	-	+	+	+
Small artisanal fishery	-	a	a	+
Recreational fishing (angling)	-	b	+	+
Spearfishing	-	-	-	+

^a^ Only for firms legally resident in the local province.

^b^ Only for residents in the local municipality.

### Sampling design

Differences in the type of substrate and location (i.e. distance from the coast) can influence the structure of the fish assemblages, and may account for much of the variability observed [48]. In the study area, the non-protected locations that may be used as control sites are present only on sandstone substrate coastal areas, while the no-take/no-entry zones are located far from the coastline, in locations with basaltic or granitic substrates. Therefore, it was not possible to compare the no-take/no-entry zones with the control sites, and two different sampling designs were implemented to evaluate the reserve effect (comparison between protected and unprotected locations) and the zonation effect (comparison among locations with different levels of protection within the MPA). To evaluate the reserve effect, two sites in zone B and two sites in zone C were compared with four control sites in four different control locations along the coastline (Fig 1). The control area is very large, and to give a clearer idea of the natural spatial variation in the characteristics of the fish assemblages, the non-protected sites were grouped into two external areas (OUT1, the closest to the boundaries of the MPA, and OUT2, which includes the two most distant control sites). Six sites (two in zone A, two in zone B, two in zone C) were also selected on the granitic substrate of Mal di Ventre Island to evaluate the zonation effect (Fig 1). All the sampling sites were exposed to the prevailing wind in the area, the Mistral, in order to reduce physical variability.

### Data collection

Four surveys were conducted in the same sampling sites in 2004, 2007, 2010 and 2012, in the period between June and July (see “Acknowledgments” section for details). Data on the abundance and size of the different fish species on the rocky bottoms of shallow waters (5 metres in depth) were collected using Underwater Visual Census (UVC). This technique has a low impact on marine ecosystems, and is therefore extensively used for monitoring MPAs [49]. Three linear transects measuring 25m x 5m were used for each site. Each transect was covered twice: the first time a speed of 5m/min was used for the census of necto-bentic and more visible species, while on the way back, a lower speed was used for the census of cryptic species [50]. UVC was always carried out by two scuba divers simultaneously: one diver video-recorded the fish fauna using an underwater video camera, while the other used a PVC slate to record the species, number and size of the individuals that were not recorded by the camera [51]. Divers were trained to standardize the estimates of fish size before beginning each survey, and in all the cases, at least one of the two operators had participated in the previous survey.

### Data analysis

Video footage was subsequently analysed by the same operators that performed the UVC. Continuous measures of total length (cm) were preferred over wider size-class estimates, in order to obtain a more accurate calculation of biomass [50]. Sizes of fish were converted to biomass values, using the weight-length conversion formula (W = aL^b^), based on information from *Fishbase* [52] referring to the closest area available. If more than one estimate for the parameters of the conversion formula was available for an area, the average was used. The conversion to biomass was performed only for commercial species, because the parameters for the length-weight relationship were not available for many non-commercial species. Gregarious and very mobile species were not included in the analysis, because mobile species are less affected by protection [53] and gregarious species present remarkably wide variability in abundance values during visual census, as a result of their schooling behaviour [54].

Two types of statistical analyses were carried out:

1. Multivariate analyses, to investigate changes in the assemblage structure across levels of protection. This type of analysis was used for the abundance of all species and for the abundance and biomass of commercial species.
2. Univariate analyses, to test for changes in the total abundance and total biomass of commercial species and separately for the sea breams *Diplodus sargus sargus* and *D*. *vulgaris* (the two main commercial species in the area).

Both types of analyses were run using permutational analysis of variance (PERMANOVA) on square root transformed data, with PRIMER6 statistical software (*Plymouth Marine Laboratory*, *UK*) complete with PERMANOVA+ package [55]. The analyses were based on Bray-Curtis dissimilarities for the multivariate technique, and on Euclidian distances for the univariate technique. Euclidian distances are a more appropriate method of partitioning when a single response variable is tested. Each term of the analysis was tested using 9999 random permutations, and was associated with a Monte Carlo test [55]. The statistical design for the reserve effect included the random factor “Year” (four levels: 2004, 2007, 2010, 2012), the fixed factor “Protection” (four levels: B, C, OUT1, OUT2) and the random factor “Site” (two levels: 1, 2) nested in “Protection”. In the design for the zonation effect, the fixed factor “Protection” included three levels (A, B, C), while the factors “Year” and “Site” were kept at the same levels used for the reserve effect design. Particular attention was paid to the interaction factor “Year x Protection”, which is able to highlight temporal changes to the response to protection among the different zones. Where this factor was statistically significant, a follow-up analysis was conducted with a pairwise test, to check which pairs of samples were significantly different. The results for the univariate analyses were represented using bar charts (mean ± standard error), while the results for the multivariate analyses were represented using two-dimensional non-metric multidimensional scaling (nMDS) plots. The similarity percentage procedure SIMPER [56] was used to identify which species contributed most to the differences between protection levels and years.

## Results

### Structure of the fish assemblage

A census was taken of fifty-five fish species belonging to 19 families (Table 2 and S1 Table for more details). In the study area, the dominant fish families in terms of abundance were Pomacentridae, Labridae, Sparidae and Serranidae. *Chromis chromis* (Pomacentridae), *Coris julis* (Labridae), *Diplodus vulgaris* and *Sarpa salpa* (Sparidae). The total number of species counted during the four surveys was higher in the MPA (51 species) than in the control areas (38 species). Within the MPA, the fish assemblage contained a higher percentage of Sparidae and a lower percentage of Labridae compared to the control areas (Table 3). Outside the MPA, some species of medium-high commercial value were absent (i.e. *Phycis phycis*, *Chelon labrosus*, *Liza aurata*, *Lithognatus mormyrus*, *Muraena helena*, *Sciaena umbra*, *Epinephelus costae*, *E*. *marginatus*, *Sphyraena viridensis*, *Dasyatis pastinaca*) (Table 2).

**Table 2:** Species composition of the fish assemblage inside and outside the Sinis MPA.

Species	MPA	OUT	Fishing value	UVC Category	Species	MPA	OUT	Fishing value	UVC Category
*Apogon imberbis*	+	+	Non comm.	B	*Liza aurata*	+	-	Comm.	A
*Atherina* sp.	+	-	Comm.	B	*Mullus surmuletus*	+	+	Comm.	A
*Parablennius gattorugine*	+	+	Non comm.	B	*Muraena helena*	+	+	Comm.	B
*Parablennius rouxi*	+	+	Non comm.	B	*Chromis chromis*	+	+	Non comm.	A
*Parablennius sanguinolentus*	+	-	Non comm.	B	*Sciaena umbra*	+	-	Comm.	A
*Parablennius zvonimiri*	+	-	Non comm.	B	*Scorpaena porcus*	+	+	Comm.	B
*Microlipophrys nigriceps*	+	-	Non comm.	B	*Scorpaena maderensis*	+	+	Comm.	B
*Seriola dumerili*	+	-	Comm.	A	*Epinephelus costae*	+	-	Comm.	A
*Spicara maena*	+	-	Comm.	A	*Epinephelus marginatus*	+	-	Comm.	A
*Phycis phycis*	+	-	Comm.	A	*Serranus cabrilla*	+	+	Comm.	A
*Gobius bucchichi*	+	+	Non comm.	B	*Serranus scriba*	+	+	Comm.	A
*Gobius cobitis*	+	-	Non comm.	B	*Boops boops*	-	+	Comm.	A
*Gobius cruentatus*	-	+	Non comm.	B	*Dentex dentex*	-	+	Comm.	A
*Gobius geniporus*	+	+	Non comm.	B	*Diplodus annularis*	+	+	Comm.	A
*Gobius paganellus*	-	+	Non comm.	B	*Diplodus puntazzo*	+	+	Comm.	A
*Coris julis*	+	+	Non comm.	A	*Diplodus sargus sargus*	+	+	Comm.	A
*Labrus merula*	+	+	Comm.	A	*Diplodus vulgaris*	+	+	Comm.	A
*Labrus viridis*	+	+	Comm.	A	*Lithognathus mormyrus*	+	-	Comm.	A
*Symphodus cinereus*	+	-	Non comm.	A	*Oblada melanura*	+	+	Comm.	A
*Symphodus doderleini*	+	-	Non comm.	A	*Sarpa salpa*	+	+	Non comm.	A
*Symphodus mediterraneus*	+	+	Non comm.	A	*Sparus aurata*	+	+	Comm.	A
*Symphodus tinca*	+	+	Non comm.	A	*Spondyliosoma cantharus*	+	+	Comm.	A
*Symphodus melanocercus*	+	+	Non comm.	A	*Sphyraena viridensis*	+	-	Comm.	A
*Symphodus ocellatus*	+	+	Non comm.	A	*Tripterygion delaisi*	+	+	Non comm.	B
*Symphodus roissali*	+	+	Non comm.	A	*Tripterygion melanurum*	+	+	Non comm.	B
*Symphodus rostratus*	+	+	Non comm.	A	*Tripterygion tripteronotum*	+	-	Non comm.	B
*Thalassoma pavo*	+	+	Non comm.	A	*Dasyatis pastinaca*	+	-	Comm.	A
*Chelon labrosus*	+	-	Comm.	A					

Reported for every species is the fishing value and the category of detectability during the visual census: (A) necto-benctic and more visible species. (B) cryptic species. (Comm.) commercial. (Non comm.) non commercial.

**Table 3:** Percentages of abundance of the fish families in the assemblages inside and outside the Sinis MPA.

Family	MPA	OUT
Apogonidae	1.18	0.46
Atherinidae	2.47	0.00
Blenniidae	0.31	0.09
Carangidae	0.25	0.00
Centracanthidae	0.04	0.00
Dasyatidae	0.01	0.00
Gadidae	0.01	0.00
Gobiidae	0.29	0.57
Labridae	33.53	42.40
Mugilidae	0.12	0.00
Mullidae	0.37	0.93
Muraenidae	0.14	0.03
Pomacentridae	32.98	37.60
Sciaenidae	0.07	0.00
Scorpaenidae	0.23	0.13
Serranidae	2.68	2.34
Sparidae	24.96	15.34
Sphyraenidae	0.05	0.00
Tripterygiidae	0.32	0.11

### Multivariate analysis

The interaction “Year x Protection” proved significant in all the multivariate analyses (Table 4). In the analyses of the abundance data for all fish species, the factors “Year” and “Site(Protection)” (S2 Table) were also significant. In 2007, the composition of the assemblage in zone B was different from the assemblage in zones C, OUT1 and OUT2 (Table 4). Differences between years were detected in zone B (2007 vs 2004 and 2012) and OUT1 (2007 vs 2004 and 2010). Zones A, B and C differed from each other in 2004 and 2007, but not in the years thereafter (Table 4). The composition of the assemblage in zone A was always different in terms of abundance over the years, with the exception of the period 2010–2012. In zone B, differences were detected when 2004 was compared with 2007 and 2012, while in zone C the only significant difference was detected between 2004 and 2007 (Table 4).

**Table 4:** Results of multivariate Permanova analyses on square root transformed data for “Year x Protection” factor. Pairwise tests are reported in the event of statistical significance.

**Permanova**						
**Analysis**	**Source**	**Variable**	**df**	**MS**	**Pseudo-F**	**Perms**
Reserve effect	All species	Abundance	9	1272.40	1.74**	9868.00
**Pairwise test**						
	**B, C**	**B, OUT1**	**B, OUT2**	**C, OUT1**	**C, OUT2**	**OUT1, OUT2**
2004	1.41	1.18	1.15	1.08	0.98	1.00
2007	2.38*	2.63*	2.20*	1.70	1.51	1.44
2010	0.80	1.09	0.76	1.32	1.49	1.31
2012	0.75	1.27	1.46	1.40	1.42	1.34
**Zone**	**2004, 2007**	**2004, 2010**	**2004, 2012**	**2007, 2010**	**2007, 2012**	**2010, 2012**
B	2.99*	1.19	1.59	1.28	2.25*	1.28
C	2.13	1.34	1.27	1.64	1.20	1.40
OUT1	2.35*	0.95	1.18	2.41*	1.55	1.44
OUT2	1.45	1.42	1.98	0.64	1.93	1.48
**Permanova**						
**Analysis**	**Source**	**Variable**	**df**	**MS**	**Pseudo-F**	**Perms**
Zonation effect	All species	Abundance	6	1113.60	2.18***	9889.00
**Pairwise test**						
**Year**	**A, B**	**A, C**	**B, C**			
2004	7.09***	3.21**	4.61***			
2007	2.17*	2.46**	2.54**			
2010	1.06	0.88	0.91			
2012	1.12	1.19	0.97			
**Zone**	**2004, 2007**	**2004, 2010**	**2004, 2012**	**2007, 2010**	**2007, 2012**	**2010, 2012**
A	3.18*	3.47*	2.98*	3.16*	2.06*	1.62
B	4.76**	2.14	3.10**	1.85	1.92	1.68
C	3.60*	1.21	1.56	1.97	1.32	1.03
**Permanova**						
**Analysis**	**Source**	**Variable**	**df**	**MS**	**Pseudo-F**	**Perms**
Reserve effect	Commercial species	Abundance	9	1550.80	1.97 **	9886.00
**Pairwise test**						
**Year**	**B, C**	**B, OUT1**	**B, OUT2**	**C, OUT1**	**C, OUT2**	**OUT1, OUT2**
2004	1.35	0.49	0.78	1.05	1.20	0.66
2007	2.19*	2.30*	2.06	1.67	0.98	1.19
2010	0.71	1.66	1.13	1.43	1.51	1.81
2012	1.12	1.32	2.01	1.38	1.76	0.81
**Zone**	**2004, 2007**	**2004, 2010**	**2004, 2012**	**2007, 2010**	**2007, 2012**	**2010, 2012**
B	3.45	1.39	1.60	1.31	2.27	1.49
C	4.80*	1.18	1.58	1.99	1.34	1.65
OUT1	3.54*	1.06	1.46	1.99	1.67	1.79
OUT2	1.28	1.43	1.76	0.68	3.86*	0.83
**Permanova**						
**Analysis**	**Source**	**Variable**	**df**	**MS**	**Pseudo-F**	**Perms**
Zonation effect	Commercial species	Abundance	6	1490.20	2.43 **	9916.00
**Pairwise test**						
**Year**	**A, B**	**A, C**	**B, C**			
2004	2.70**	1.82*	2.54**			
2007	1.68	2.18*	2.19**			
2010	1.81*	1.05	1.42			
2012	1.01	1.72*	1.50			
**Zone**	**2004, 2007**	**2004, 2010**	**2004, 2012**	**2007, 2010**	**2007, 2012**	**2010, 2012**
A	1.98	2.10*	1.59	3.14*	3.08*	1.19
B	2.59*	2.06	2.04*	1.64	1.23	1.43
C	4.67**	1.38	1.20	1.10*	1.23	0.54
**Permanova**						
**Analysis**	**Source**	**Variable**	**df**	**MS**	**Pseudo-F**	**Perms**
Reserve effect	Commercial species	Biomass	9	2131.00	2.15 **	9899.00
**Pairwise test**						
**Year**	**B, C**	**B, OUT1**	**B, OUT2**	**C, OUT1**	**C, OUT2**	**OUT1, OUT2**
2004	1.04	0.74	0.81	1.01	1.17	0.73
2007	1.63	3.44**	2.83*	2.25	1.68	1.52
2010	0.72	1.14	0.98	1.12	1.55	1.85
2012	1.30	1.91*	2.35**	1.50	1.30	0.99
**Zone**	**2004, 2007**	**2004, 2010**	**2004, 2012**	**2007, 2010**	**2007, 2012**	**2010, 2012**
B	2.72*	1.11	1.31	1.30	3.11*	1.13
C	21.25***	1.11	1.18	1.75	0.97	1.53
OUT1	3.75*	1.06	1.64	2.15	2.77*	1.57
OUT2	1.15	1.50	1.35	1.16	6.74**	1.08
**Permanova**						
**Analysis**	**Source**	**Variable**	**df**	**MS**	**Pseudo-F**	**Perms**
Zonation effect	Commercial species	Biomass	6	1808.60	1.79 *	9917.00
**Pairwise test**						
**Year**	**A, B**	**A, C**	**B, C**			
2004	3.72**	2.42*	5.32**			
2007	2.67*	8.94**	2.15*			
2010	0.89	1.11	0.99			
2012	1.02	1.18	1.12			
**Zone**	**2004, 2007**	**2004, 2010**	**2004, 2012**	**2007, 2010**	**2007, 2012**	**2010, 2012**
A	0.64	1.71	1.22	1.90	1.93	1.12
B	1.77	1.63	1.91	1.33	1.13	1.56
C	2.93*	1.54	1.45	1.36	1.22	1.00

*p<0.05

** p<0.01

*** p<0.001.

**Fig 2:**
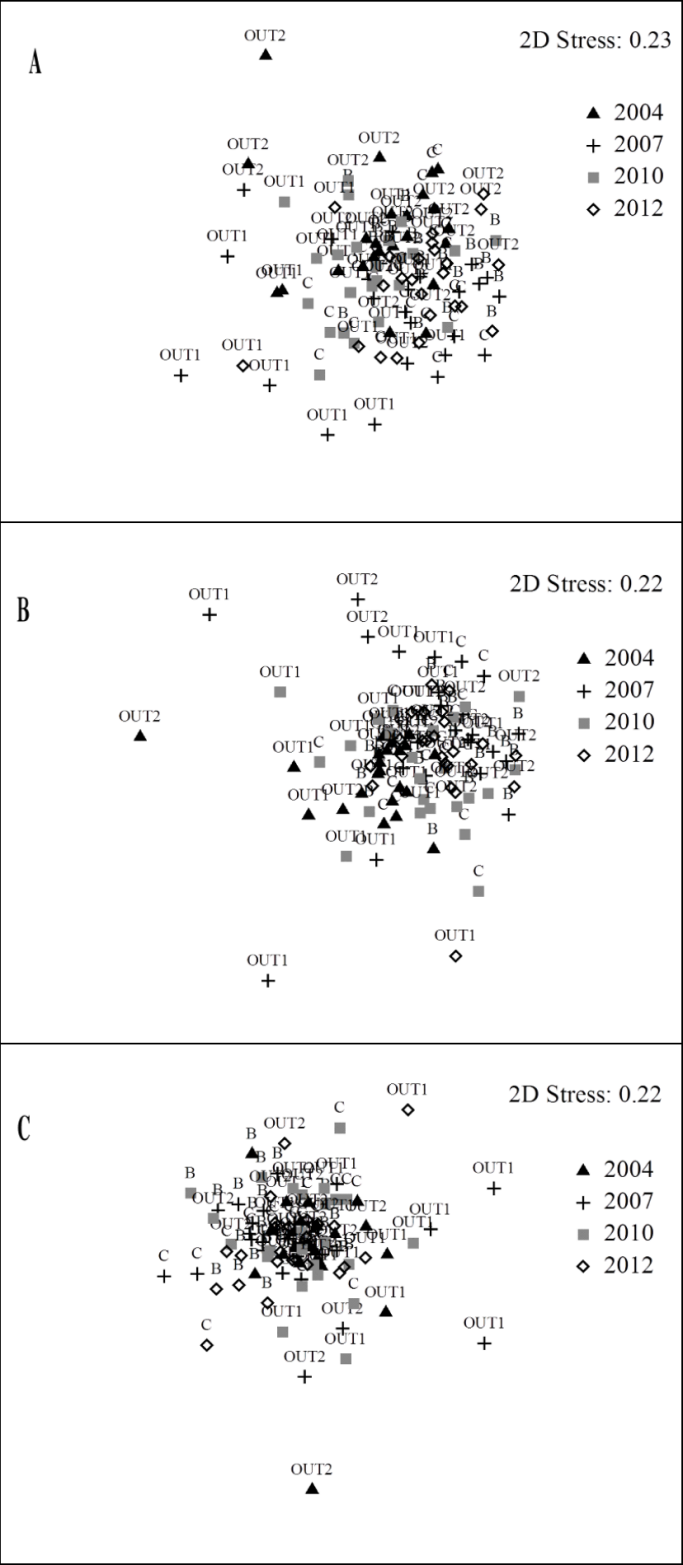
Reserve effect. **Two-dimensional nMDS plots, in the different zones in the Sinis MPA and control locations.** (A) abundance of the entire assemblage; (B) abundance of commercial species; (c) biomass of commercial species.

**Fig 3:**
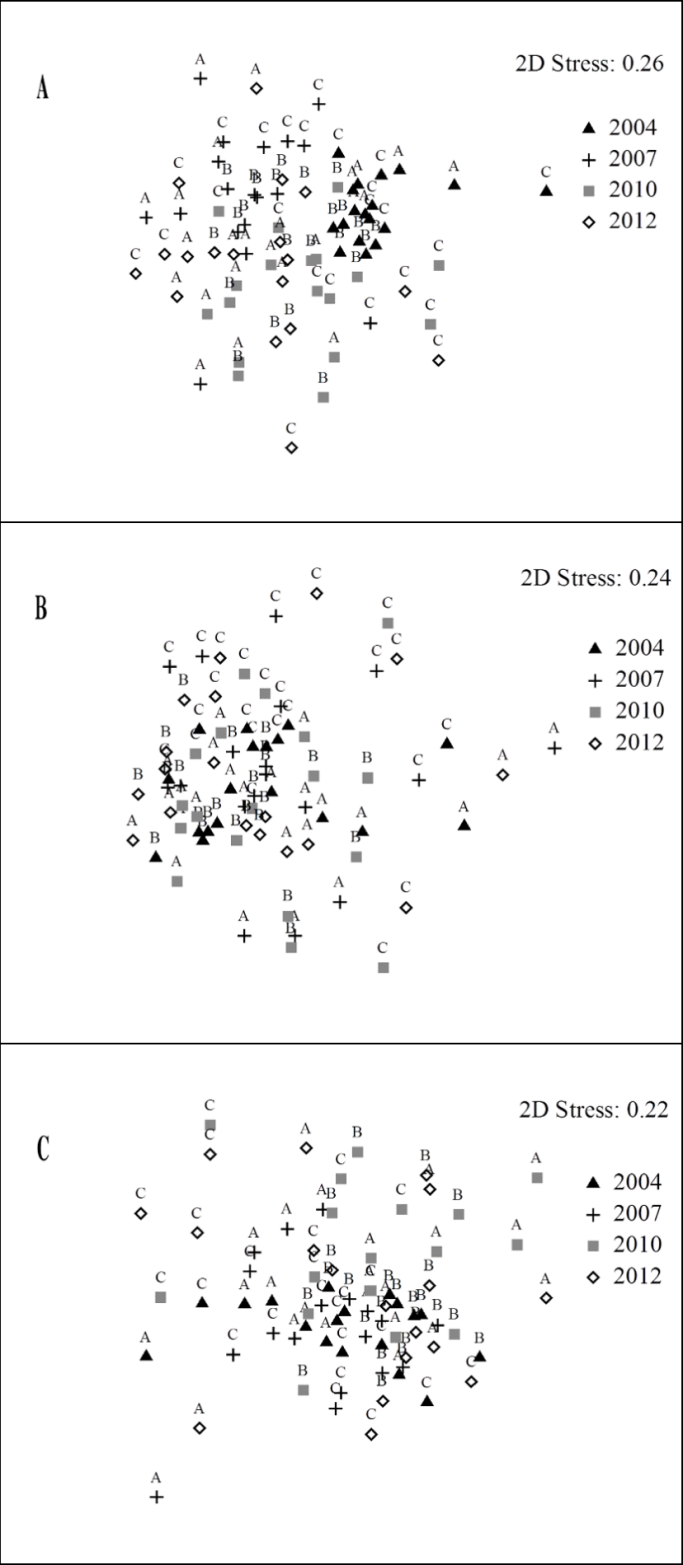
Zonation effect. **Two-dimensional nMDS plots for the different zones in the Sinis MPA.** (A) abundance of the entire assemblage; (B) abundance of commercial species; (C) biomass of commercial species.

These dissimilarities were mainly determined by variations in the abundance of *Coris julis*, with a strong contribution also made by *D*. *sargus sargus*, *D*. *vulgaris* and *Symphodus tinca* (S3 Table). However, with the exception of significant differences regarding the year 2007, no clear patterns of variation emerged from the analysis of the reserve effect or the zonation effect, and samples from different years and levels of protection were extremely mixed in the nMDS plot (Figs 2A and 3A).

The multivariate PERMANOVA run on the abundance data regarding commercial fish species showed that the factors “Year” and “Site(Protection)” were significant (S2 Table). When the effect of zonation was tested, “Year” and “Protection” also proved significant (S2 Table). In terms of abundance, the composition of commercial species in zone B was different from zones C and OUT1 in 2007 (Table 4). Significant differences between years were also detected in zones C, OUT1 and OUT2, regarding the year 2007 in all cases. In the analyses regarding the effect of zonation, zones A, B and C differed from each other in 2004, while in 2007, zone C differed from A and B. In 2010, zone A differed from B, and in 2012, zone A differed from C. Between years, the composition of commercial species varied in some cases within zones (Table 4). Also for commercial species, the high overlap in the samples in the nMDS plots showed the lack of a clear trend of diversification among zones, in the analysis regarding both the reserve and the zonation effect (Figs 2B and 3B).

The analysis of biomass data to test for the reserve effect showed that the significant factors other than “Year x Protection”, affecting the composition of commercial species, were “Year” and “Site(Protection)” (S1 Table). When the effect of zonation was tested, “Year” was also significant (S1 Table). The composition of commercial species in B was different from OUT1 and OUT2 in 2007 and 2012 (Table 4). The differences between years detected within each zone regarded the year 2007 in all cases. When the effect of zonation was tested, zones A, B and C differed from each other in 2004 and 2007, but not in 2010 and 2012. The three protection zones did not display significant inter-annual variations, with the sole exception of zone C, in which the biomass values of commercial species were different in 2004 compared to 2007 (Table 4). The multivariate analyses of the biomass data of commercial species did not detect clear patterns of variation either, and samples in the nMDS plots were extremely mixed (Figs 2C and 3C).

### Univariate analysis

**Fig 4:**
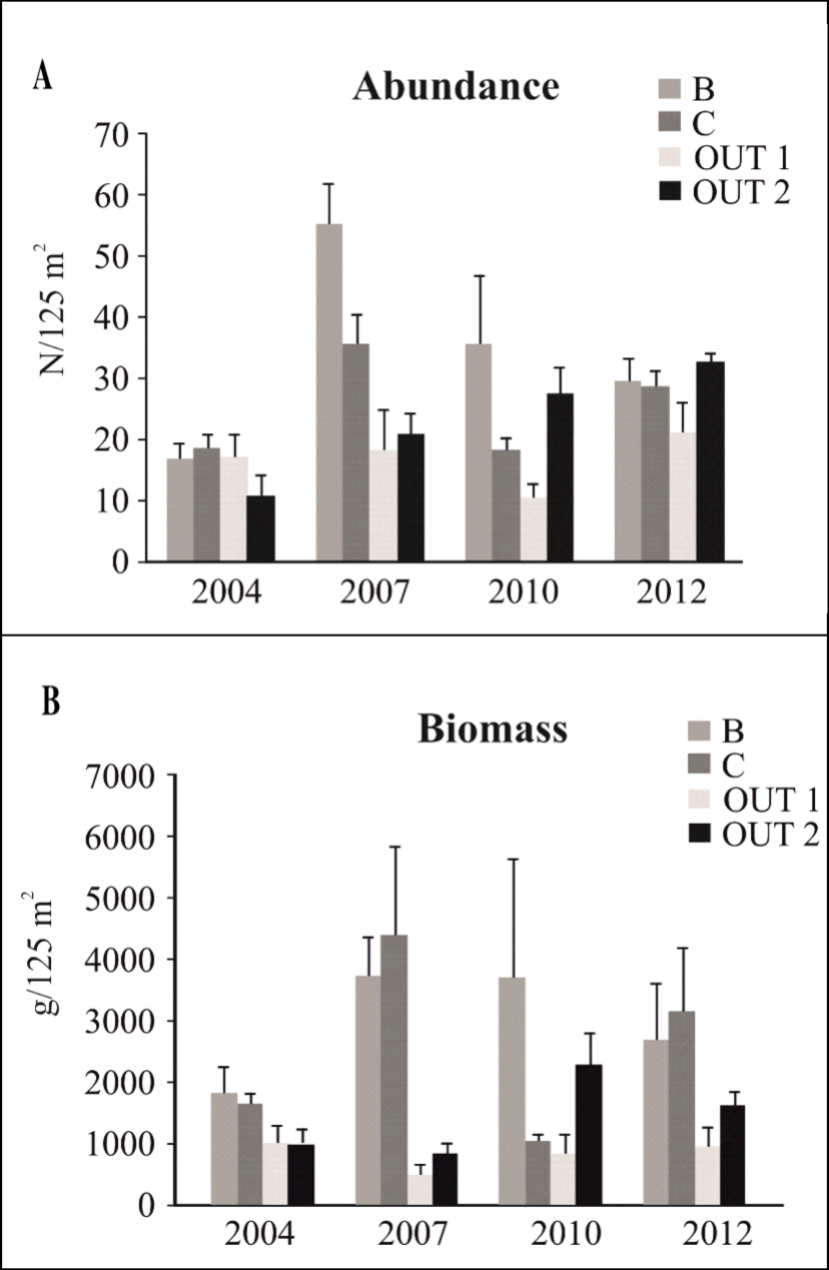
Reserve effect on commercial species. Mean values (± SE) of (A) abundance and (B) biomass.

**Fig 5:**
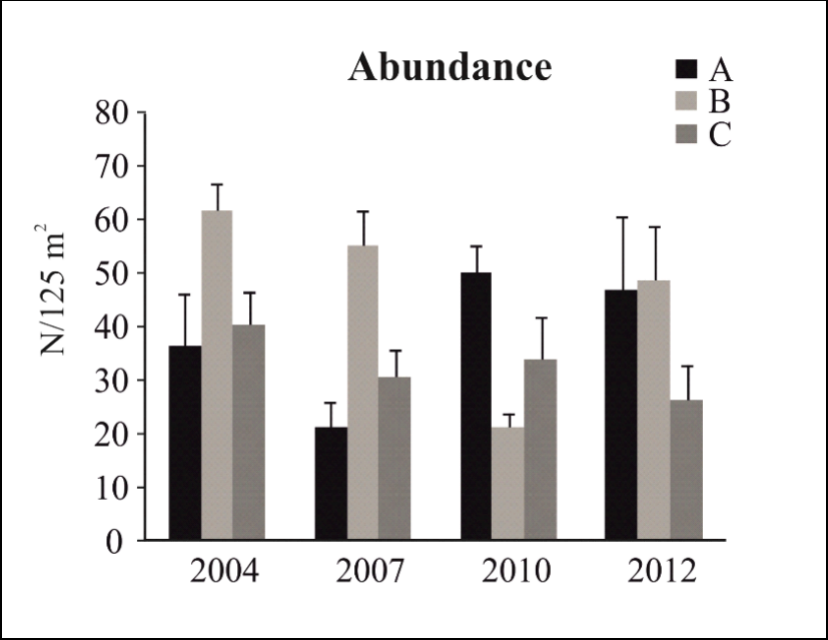
Zonation effect on commercial species. Mean values (± SE) of abundance.

The analysis of the total abundance of commercial species showed that the interaction “Year x Protection” was significant both for the reserve effect and the zonation effect (Table 5). “Year” was also a significant factor when the reserve effect was tested (S4 Table). Differences in the total abundance of commercial species between the protected and unprotected sites were significant only in 2007, when zone B showed higher values than OUT2 (Table 5 and Fig 4A). Some significant differences between years were detected in zone B and C, while the inter-annual variations observed in the control zones were never significant (Table 5). In zone B, total abundance increased during the period 2004–2007 and then decreased constantly (Fig 4A), but these variations were not significant (Table 5). In 2012 the abundance value of zone B was higher than in 2004. In zone C, total abundance increased in 2007 compared to 2004, but in the following three years it decreased to the initial level, before rising again in the period 2010–2012 (Fig 4A). In the analysis regarding the effect of zonation, significant differences in total abundance were detected only in 2004, when zone B had higher levels than both zone C and zone A (Table 5 and Fig 5). Few differences between years were detected: the inter-annual variations observed in zone A were never significant, while a significant decrease in total abundance was detected in zone B in2004-2010 and in zone C in 2010–2012 (Table 5 and Fig 5).

**Table 5:** Results of univariate Permanova analyses on square root transformed data for “Year x Protection” factor for commercial species. Pairwise tests are reported in the event of statistical significance.

**Permanova**						
**Analysis**	**Source**	**Variable**	**df**	**MS**	**Pseudo-F**	**Perms**
Reserve effect	Commercial species	Abundance	9	4.09	2.96 *	9949.00
**Pairwise test**						
	**B, C**	**B, OUT1**	**B, OUT2**	**C, OUT1**	**C, OUT2**	**OUT1, OUT2**
2004	0.71	0.07	0.87	0.59	1.15	0.79
2007	3.11	3.31	4.44*	1.96	2.30	0.56
2010	1.38	2.43	0.46	1.55	1.32	2.80
2012	0.12	2.47	1.00	1.59	0.78	5.25
**Zone**	**2004, 2007**	**2004, 2010**	**2004, 2012**	**2007, 2010**	**2007, 2012**	**2010, 2012**
B	5.15	2.54	33.99*	1.36	2.90	0.42
C	15.22*	0.09	1.49	2.13	0.73	88.94**
OUT1	0.24	9.43	2.17	1.60	0.74	4.53
OUT2	2.94	1.31	2.88	0.63	2.82	0.90
**Permanova**						
**Analysis**	**Source**	**Variable**	**df**	**MS**	**Pseudo-F**	**Perms**
Zonation effect	Commercial species	Abundance	6	9.45	3.94 *	9960.00
**Pairwise test**						
**Year**	**A, B**	**A, C**	**B, C**			
2004	11.98**	2.66	26.05**			
2007	3.48	2.69	2.13			
2010	4.13	1.18	0.91			
2012	0.48	1.11	1.63			
**Zone**	**2004, 2007**	**2004, 2010**	**2004, 2012**	**2007, 2010**	**2007, 2012**	**2010, 2012**
A	10.52	3.10	0.71	4.88	2.50	0.45
B	0.51	24.09*	3.19	2.75	0.44	13.26
C	2.58	0.58	1.12	0.11	0.32	68.96**
**Permanova**						
**Analysis**	**Source**	**Variable**	**df**	**MS**	**Pseudo-F**	**Perms**
Reserve effect	Commercial species	Biomass	9	608.32	4.06 *	9962.00
**Pairwise test**						
**Year**	**B, C**	**B, OUT1**	**B, OUT2**	**C, OUT1**	**C, OUT2**	**OUT1, OUT2**
2004	0.19	1.75	1.53	5.39*	1.93	0.35
2007	0.37	7.29*	17.17**	5.26*	5.51*	1.47
2010	2.03	1.96	0.50	0.62	2.31	1.88
2012	0.49	6.13*	3.02	2.61	1.44	7.17*
**Zone**	**2004, 2007**	**2004, 2010**	**2004, 2012**	**2007, 2010**	**2007, 2012**	**2010, 2012**
B	2.81	2.80	2.13	0.72	3.44	0.58
C	3.07	2.92	1.59	7.06	0.61	1.94
OUT1	2.72	0.57	11.42	2.22	2.35	0.32
OUT2	0.15	1.50	1.83	2.38	6.05	1.15
**Permanova**						
**Analysis**	**Source**	**Variable**	**df**	**MS**	**Pseudo-F**	**Perms**
Zonation effect	Commercial species	Biomass	6	2040.00	2.31	9950.00

*p<0.05

** p<0.01.

When the total biomass of commercial species was considered, the interaction “Year x Protection” was significant only in the analysis regarding the reserve effect. “Protection” was also a significant factor (S4 Table). No significant factors were detected when the zonation effect was tested. In 2007, the total biomass of commercial species was higher in zone B and C than in the control sites, while in 2012, zone B presented higher values compared to OUT1 and significant differences were also detected between the two control zones (Table 5 and Fig 4B). No significant differences were observed between years in the different protection zones.

**Fig 6:**
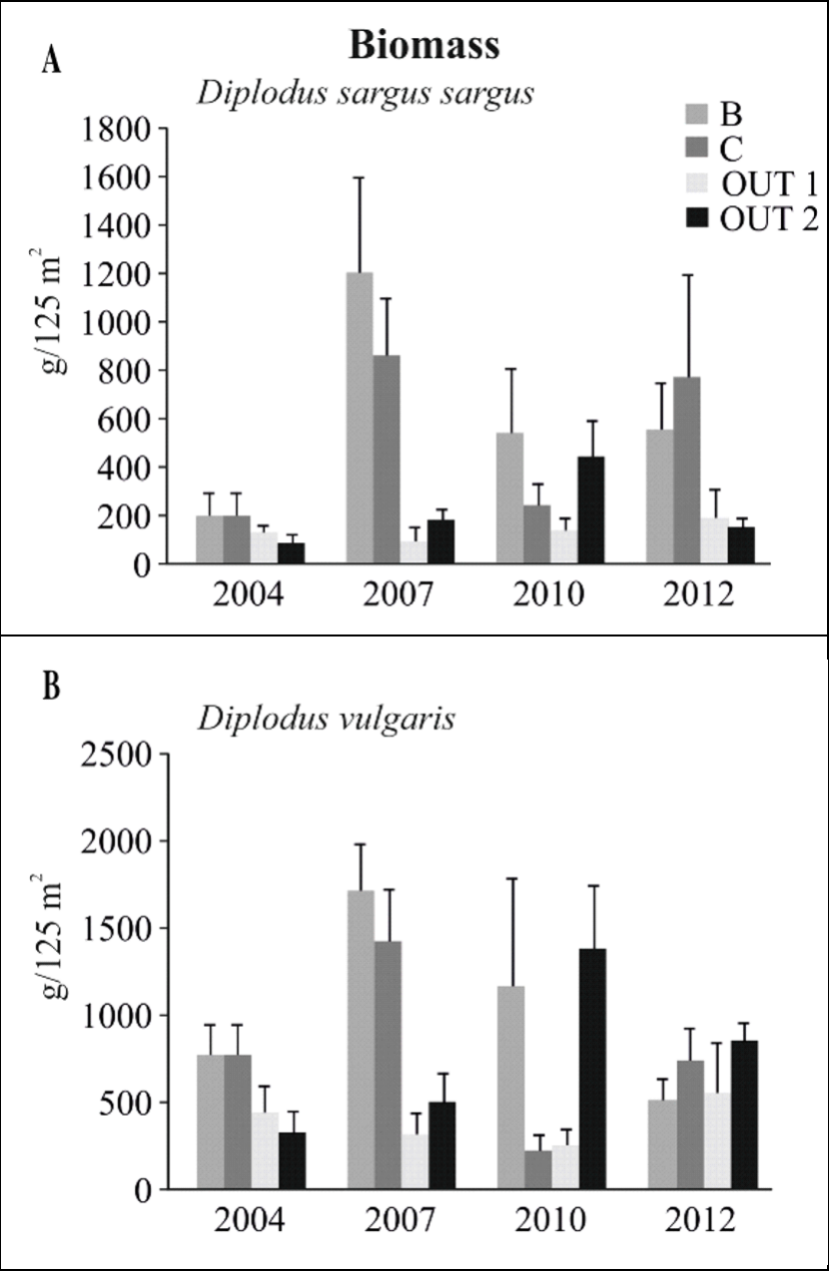
Reserve effect on the two main commercial species in the area. (A) *Diplodus sargus sargus*.(B) *Diplodus vulgaris*. Mean values (± SE) of biomass.

In the analysis of the reserve effect on the two most representative commercial species in the area, *D*. *sargus sargus* and *D*. *vulgaris*, the interaction “Year x Protection” was significant for biomass data (Table 6) and only close to significance for abundance (*D*. *sargus sargus*: p = 0.052; *D*. *vulgaris*: p = 0.053). In both species, the only significant factor affecting abundance was “Year” (S4 Table). This factor was also significant in the test for the reserve effect on biomass of *D*. *sargus sargus* (S4 Table). No significant factors were detected for these species when the zonation effect on their values of abundance and biomass was tested (Table 6). *Diplodus sargus sargus* had a significantly higher biomass in zone B in comparison with OUT1 and OUT2 in 2007, and with OUT2 in 2012 (Table 6 and Fig 6A). Between years, the only difference was detected in B, with biomass in 2007 higher than in 2012 (Table 6 and Fig 6A). In 2007, zone B also presented a significantly higher biomass of *D*. *vulgaris* than OUT1 and OUT2. The tests between years for this species showed that in zone C, biomass increased in the period 2010–2012. An increase in biomass was also detected in OUT2, where the level in 2012 was higher than in 2007 (Table 6 and Fig 6B).

**Table 6:** Results of univariate Permanova analyses on square root transformed data for “Year x Protection” factor for *Diplodus* species. Pairwise tests are reported in the event of statistical significance.

**Permanova**						
**Analysis**	**Source**	**Variable**	**df**	**MS**	**Pseudo-F**	**Perms**
Reserve effect	*D*. *sargus sargus*	Abundance	9	3.30	2.76	9952.00
**Analysis**	**Source**	**Variable**	**df**	**MS**	**Pseudo-F**	**Perms**
Reserve effect	*D*. *sargus sargus*	Biomass	9	188.46	2.78 *	9958.00
**Pairwise test**						
**Year**	**B, C**	**B, OUT1**	**B, OUT2**	**C, OUT1**	**C, OUT2**	**OUT1, OUT2**
2004	0.06	0.33	1.16	0.39	1.72	0.90
2007	0.71	5.08 *	19.44 **	2.94	2.72	1.37
2010	0.63	1.10	0.05	1.74	1.44	3.05
2012	0.43	2.48	7.44 *	2.02	2.50	0.15
**Zone**	**2004, 2007**	**2004, 2010**	**2004, 2012**	**2007, 2010**	**2007, 2012**	**2010, 2012**
B	7.16	0.60	4.04	1.20	34.27 *	0.22
C	4.13	2.68	3.60	4.47	8.28	3.85
OUT1	2.21	0.33	0.58	0.77	6.98	0.41
OUT2	2.55	2.25	1.60	2.01	2.63	3.08
**Permanova**						
**Analysis**	**Source**	**Variable**	**df**	**MS**	**Pseudo-F**	**Perms**
Zonation effect	*D*. *sargus sargus*	Abundance	6	3.40	3.10	9950.00
**Analysis**	**Source**	**Variable**	**df**	**MS**	**Pseudo-F**	**Perms**
Zonation effect	*D*. *sargus sargus*	Biomass	6	181.91	1.39	9947.00
**Analysis**	**Source**	**Variable**	**df**	**MS**	**Pseudo-F**	**Perms**
Reserve effect	*D*. *vulgaris*	Abundance	9	3.46	2.74	9938.00
**Analysis**	**Source**	**Variable**	**df**	**MS**	**Pseudo-F**	**Perms**
Reserve effect	*D*. *vulgaris*	Biomass	9	463.90	3.59 *	9944.00
**Pairwise test**						
**Year**	**B, C**	**B, OUT1**	**B, OUT2**	**C, OUT1**	**C, OUT2**	**OUT1, OUT2**
2004	0.00	1.05	1.41	1.07	1.44	0.36
2007	0.68	4.82 *	4.79 *	2.81	2.48	0.63
2010	2.09	2.06	0.84	0.15	3.10	3.15
2012	0.66	1.02	1.62	1.04	0.34	2.37
**Zone**	**2004, 2007**	**2004, 2010**	**2004, 2012**	**2007, 2010**	**2007, 2012**	**2010, 2012**
B	6.97	2.45	0.75	3.55	4.26	1.01
C	5.11	1.42	0.05	2.02	0.84	36.77 *
OUT1	1.33	2.21	0.28	2.67	1.24	1.38
OUT2	1.19	1.74	3.61	1.99	50.55 *	0.84
**Permanova**						
**Analysis**	**Source**	**Variable**	**df**	**MS**	**Pseudo-F**	**Perms**
Zonation effect	*D*. *vulgaris*	Abundance	6	9.24	3.28	9945.00
**Analysis**	**Source**	**Variable**	**df**	**MS**	**Pseudo-F**	**Perms**
Zonation effect	*D*. *vulgaris*	Biomass	6	968.98	2.66	9954.00

*p<0.05

** p<0.01.

## Discussion

Marine protected areas are an example of an ecosystem-based approach to conservation, as they allow for the protection not only of the target species, but of the whole marine community. For this reason, MPAs have been advocated as powerful conservation tools that allow for the recovery of impacted areas and stocks subjected to excessive fishing pressure, thus promoting biodiversity [57]. The number of MPAs has significantly increased in the recent past (and many more are to be established), with the principal aim of tackling the failure of previous single-species conservation measures to stem the decline of marine populations[58]. Despite the prevailing enthusiasm of the scientific community concerning the effectiveness of MPAs as a conservation tool, in many cases the MPAs have had little effect on marine biological resources, and performance has been poor [44, 59].

This work has presented yet another study of an underperforming MPA. If we consider the reserve effect, although the interaction “Year x Protection” was significant in the univariate and multivariate analyses, the pattern of variation in abundance and biomass of fish species (both of all species and of commercial fish only) that emerged is often unclear. Furthermore, the results of the analyses regarding the effect of zonation did not highlight a strong response to protection, and the differences initially detected among zones A, B and C were not detected in the surveys conducted subsequently. Some large commercial species (e.g. the dusky grouper *Epinephelus marginatus*, the goldblotch grouper *E*. *costae* or the brown meagre *Sciaena umbra*) were found only within the MPA, though their abundance levels were extremely low (less than 10 individuals per year), indicating consistently unsustainable fishing pressure, even within the MPA. When compared to other Mediterranean MPAs after a similar period of protection (e.g. [60, 61]), the Sinis MPA showed poorer results for these species. A positive effect of the MPA emerged regarding the most abundant commercial fish family, Sparidae, which showed a higher number of individuals in the MPA than in the control areas. In contrast, Labridae (mainly represented by the Mediterranean rainbow wrasse: *Coris julis*) were more abundant outside the MPA. Because of the very low abundance of piscivorous species, differences in predation pressure among the control and protected locations (resulting in cascade effects on prey abundances) [62] are unlikely to explain these results, and competition for food resources seems a more likely factor. *C*. *julis* feeds on invertebrates, such as sea urchins [63], which are also an important part of the diet of commercial species such as *Diplodus sargus sargus*, *D*. *vulgaris* and *Sparus aurata* [62, 64]. In the study area, the use of gillnets is common among local fishermen, and sea breams are a typical target of this kind of fishing. Gillnet fishing does not have a major impact on *C*. *julis*, which can easily escape from the mesh of the gillnets due to its small size. The highest proportion of Labridae in the unprotected assemblages could therefore be an indication of the different fishing pressures affecting the two families, and the consequent reduction in competition for food resources for *C*. *julis*.

Responses to protection can occur in a short period of time for species with a high growth rate, high reproductive output, early age and small size at sexual maturity [35]. Because the two most abundant commercial species in the study area (*D*. *sargus sargus*, *D*. *vulgaris*) reach sexual maturity at 4 years old [52], differences among protected and unprotected areas were already expected during the first survey (conducted seven years after the establishment of the Sinis MPA). However, in the data from the first survey, no appreciable differences were evident between the protected and unprotected sites, or among zones A, B and C. When some significant differences in levels of protection were observed, zone A presented even lower levels of abundance and biomass than the buffer zones. A long-term study conducted on two MPAs in the Philippines demonstrated that the re-opening to fishery of protected areas can quickly nullify the results of years of protection [34]. The redefinition of zonation in the Sinis MPA, which occurred in 2003, might therefore have had a significant impact on the results obtained in the 2004 survey.

Within the timeframe of this study, most of the differences in species abundance and biomass between the protected and unprotected sites regarded the year 2007. However, the analysis of temporal patterns of variation in abundance and biomass reveals that these metrics increased and decreased randomly across the years, both in the protected sites and in the control sites. Thus, it is unlikely that the results of 2007 are the effect of the protection measures applied in the MPA.

Moreover, “Year” and “Site(Protection)” were significant factors in the multivariate PERMANOVA, highlighting the importance of intrinsic temporal and spatial variability for assemblage composition. Fluctuations in the abundance and biomass of commercial species are common both in protected and unprotected areas [32, 36], and therefore the occasional rise of differences is not necessarily evidence of the species’ response to protection.

Temporal trends in commercial species showed that the Sinis MPA did not provide an adequate level of protection, even if we consider the changes to zonation carried out ten years ago, which might have nullified any positive effects of protection existing until 2003. It is often pointed out that the partial protection in the buffer areas might produce variable responses in the marine fauna: in some cases, buffer areas played a part in the recovery of exploited stocks [27, 28] while in other cases no positive responses were detected in comparison with the unprotected locations [26, 65]. The recovery of exploited stocks in MPAs depends to a large extent on the strength of the fishing regulations in place. Thus, the lack of a substantial difference in stock recovery between the protected sites and the control sites in the case study area may be attributed to the fact that the MPAs' fishing regulations are not significantly more restrictive than the fishing regulations in force outside the MPA. Furthermore, the large size of the Sinis MPA makes it difficult to combat poaching. Episodes of illegal fishing were often noted during monitoring activities, especially regarding the practice of spearfishing (for examples of the effects of spearfishing on fish communities, see [66, 67]). Inadequate enforcement levels are a key issue affecting several underperforming MPAs [45,46] and the Sinis MPA was expressly described as a “paper park”, i.e. only virtually protected, in a recent work [44]. The low enforcement levels in the area were also indicated as the main reason for the failure of stock re-building for other species, such as *Paracentrotus lividus* [68] or *Patella ferruginea* [69]. Considering the large size of the MPA and the relatively high number of professional and recreational fishermen operating in the area (there are about 400 fishing vessels in the area, 115 of them authorized to operate within the boundaries of the MPA), improving surveillance and enforcement is essential in order to make the MPA effective and to meet the ecological objectives for which it was established.

The analyses of the temporal pattern suggest that results are driven by factors other than the level of legal protection. In most cases, a significant differentiation in the metrics analysed regarded zone B, in comparison to controls when the reserve effect was tested, as in this zone the abundance and biomass of species tended to be more stable over the years. This may be due to the limited accessibility of zone B (difficult to access by land because of the presence of high cliffs), which might result in lower fishing pressure than in the other zones. In zone C, in contrast, where a tourist spot and a boat ramp are present, human presence is high, and this may explain the wide fluctuations in the metrics considered, which were even more intense than in the control zones. The importance of accessibility has been raised in previous studies regarding the Sinis MPA [69] and other MPAs affected by similar issues of low enforcement (e.g. [70]).

The results of this study also question the adequacy of the current zonation of the Sinis MPA. Future studies may find it useful to re-consider whether the current zonation plan is adequate to achieve the biological goals of the MPA, given the presence of no-take/no-entry zones in isolated locations with peculiar geological characteristics, which are not representative of the habitat features of the Sinis Peninsula. Islands often host different biological communities from the mainland and are therefore often designated as MPAs [71]. Within the Sinis MPA, the Island of Mal di Ventre deserves to be protected because it hosts a population of the endangered limpet *P*. *ferruginea* [69] and other species such as *Pinna nobilis*, *P*.*rudis*, *Lithophyllum* sp. and *Cystoseira* sp., included in the SPA/BIO protocol of the Barcelona Convention [72]. Regarding the fish fauna, the isolated position of the no-take/no-entry zones may limit their positive impact on other areas, since the distance from the mainland could, for example, limit spill-over [71]. Drawing on the results of the present study, the area of Torre Seu (currently included in zone B) might be suggested as a useful candidate to become a no-take/no-entry area, not only because it may be easier for enforcement bodies to monitor, but also because it might offer benefits in terms of spill-over and larval supply to the surrounding areas [41, 73]. Specific studies and monitoring should be conducted to confirm this hypothesis.

To sum up, this study has demonstrated that the ineffectiveness of this MPA in recovering exploited stocks is not attributable to a single factor, and is the result of interrelated problems concerning enforcement, regulation and spatial planning. Although MPAs have been used to a growing extent in recent years to combat the overwhelming problem of overfishing in marine ecosystems, the ecological goals for which many MPAs were established have often not been achieved. It is important to report this type of results, to demonstrate that many MPAs are only providing the illusion of conservation, and are effectively inefficient. This study therefore reinforces the existing literature on paper parks, by emphasizing the importance of multi-annual studies and the consideration of interrelated variables in assessing the effectiveness of MPAs.

## Supporting Information

S1 Table Complete dataset. (XLSX)

S2 Table Complete results of multivariate Permanova analyses on square root transformed data. *p<0.05;** p<0.01;*** p<0.001.
(XLSX)

S3 Table Results of SIMPER analyses. Fish taxa mostly contributing to the dissimilarity between years and levels of protection are shown. Only dissimilarity contributions >5% are reported.
(XLSX)

S4 Table Complete results of univariate Permanova analyses on square root transformed data. *p<0.05;** p<0.01;*** p<0.001.
(XLSX)
